# Diagnostic sensitivity and cost per diagnosis of ambulatory cardiac monitoring strategies in unexplained syncope patients

**DOI:** 10.1371/journal.pone.0270398

**Published:** 2022-06-24

**Authors:** John D. Rogers, Lucas Higuera, Sarah C. Rosemas, Ya-Jian Cheng, Paul D. Ziegler

**Affiliations:** 1 Scripps Clinic, Cardiac Pacing and Tachyarrhythmia Device Therapy, La Jolla, CA, United States of America; 2 Medtronic Inc, Cardiac Rhythm & Heart Failure, Mounds View, MN, United States of America; Universita degli Studi di Napoli Federico II, ITALY

## Abstract

Diagnosing cardiac pauses that could produce syncopal episodes is important to guide appropriate therapy. However, the infrequent nature of these episodes can make detection challenging with conventional monitoring (CM) strategies with short-term ECG monitors. Insertable cardiac monitors (ICMs) continuously monitor for arrhythmias but present a higher up-front cost. It is not well understood whether these higher costs are offset by the costs of repeat evaluation in CM strategies. We simulated the likelihood of diagnostic success and cost-per-diagnosis of pause arrhythmias with CM strategies compared to ICM monitoring. ICM device data from syncope patients diagnosed with pause arrhythmias was utilized to simulate patient pathways and diagnostic success with CM. We assumed that detected true pause episodes (≥5 seconds) were symptomatic and prompted a hospital encounter and further evaluation with CM. Subsequent true pause episodes in yet-undiagnosed patients triggered additional rounds of CM. Costs of monitoring were accrued at each encounter and represent the U.S. payer perspective. Cost per diagnosed patient was calculated as the total costs accrued for all patients divided by the number of patients diagnosed, across 1,000 simulations. During a mean 505±333 days of monitoring ICM detected 2.4±2.7 pause events per patient, with an average of 109±94 days until the first event. CM was projected to diagnose between 13.8% (24-hour Holter) and 30.2% (two 30-day monitors) of the ICM-diagnosed patients. Total diagnostic costs per ICM-diagnosed patient averaged $7,847, whereas in the CM strategies average cost-per-diagnosis ranged from $12,950±2,589 with 24-hour Holter to $32,977±14,749 for two 30-day monitors. Relative to patients diagnosed with pause arrhythmias via ICM, CM strategies diagnose fewer patients and incur higher costs per diagnosed patient.

## Introduction

Syncope is experienced by over 1 in 3 people during their lifetime [1], and carries important implications for quality of life [2] and increased risk of physical trauma and cardiovascular risks [3]. Approximately 25% of unexplained syncope patients will be diagnosed with a cardiac arrhythmia after long-term cardiac monitoring with an insertable cardiac monitor (ICM) [4]. As cardiac syncope doubles the risk of death [5], the correct identification of the etiology of syncope events is key for the provision of adequate and timely care [6]. The gold standard for the diagnosis of syncope cause is the recording of an electronic cardiography (ECG) during the syncopal event; however, the infrequent and intermittent nature of syncopal recurrence makes diagnosis challenging [6].

Health resource use associated with the evaluation of syncope is high, representing approximately 740,000 emergency department (ED) visits and 460,000 hospital admissions in the U.S. per year (representing 2% of all hospital admissions from the ED), with an annual cost estimated to exceed $2.4 billion per year [7]. Patients with unexplained syncope often incur repeated evaluations for syncope; in one study in the UK, unexplained syncope patients had seen three different specialists and underwent a median of 13 diagnostic tests for diagnostic evaluation prior to the placement of an ICM [8], with only 12% of patients receiving tests within the current guideline recommendations [9]. Indeed, an analysis as part of the Choosing Wisely campaign found that 33–44% of patients received a low-value diagnostic test after a syncopal event [10], with levels of testing utilization for syncope patients increasing between 2006–2014 [11]. In randomized trials comparing diagnostic yields of long-term continuous cardiac monitoring with an ICM versus standard of care in patients with unexplained syncope after negative initial evaluation, ICM led to an approximately 3.6 times greater rate of diagnosis (46% vs. 12%, p = 0.001) [6]. Likewise, ICMs have been shown to demonstrate a significantly higher diagnostic yield compared to short-term ECG monitors (e.g. Holter monitors and external loop recorders) due to the continuous nature of the recording and ability to capture infrequent episodes [12–14]. The present study leverages real-world data from patients with long-term ICM monitoring for unexplained syncope to simulate the relative likelihood of diagnostic success with conventional monitoring (CM) strategies using short-term ECG monitors (ranging from 24-hours to 30-days) upon syncopal recurrence, and the diagnostic cost per diagnosis of each strategy compared with ICM monitoring. Previous studies have successfully used simulation methods to analyze diagnostic techniques [15–17]. We expect that this study provides additional evidence that enables clinicians to make informed decisions about strategies to diagnose patients with cardiovascular syncope events.

## Methods

**Fig 1:**
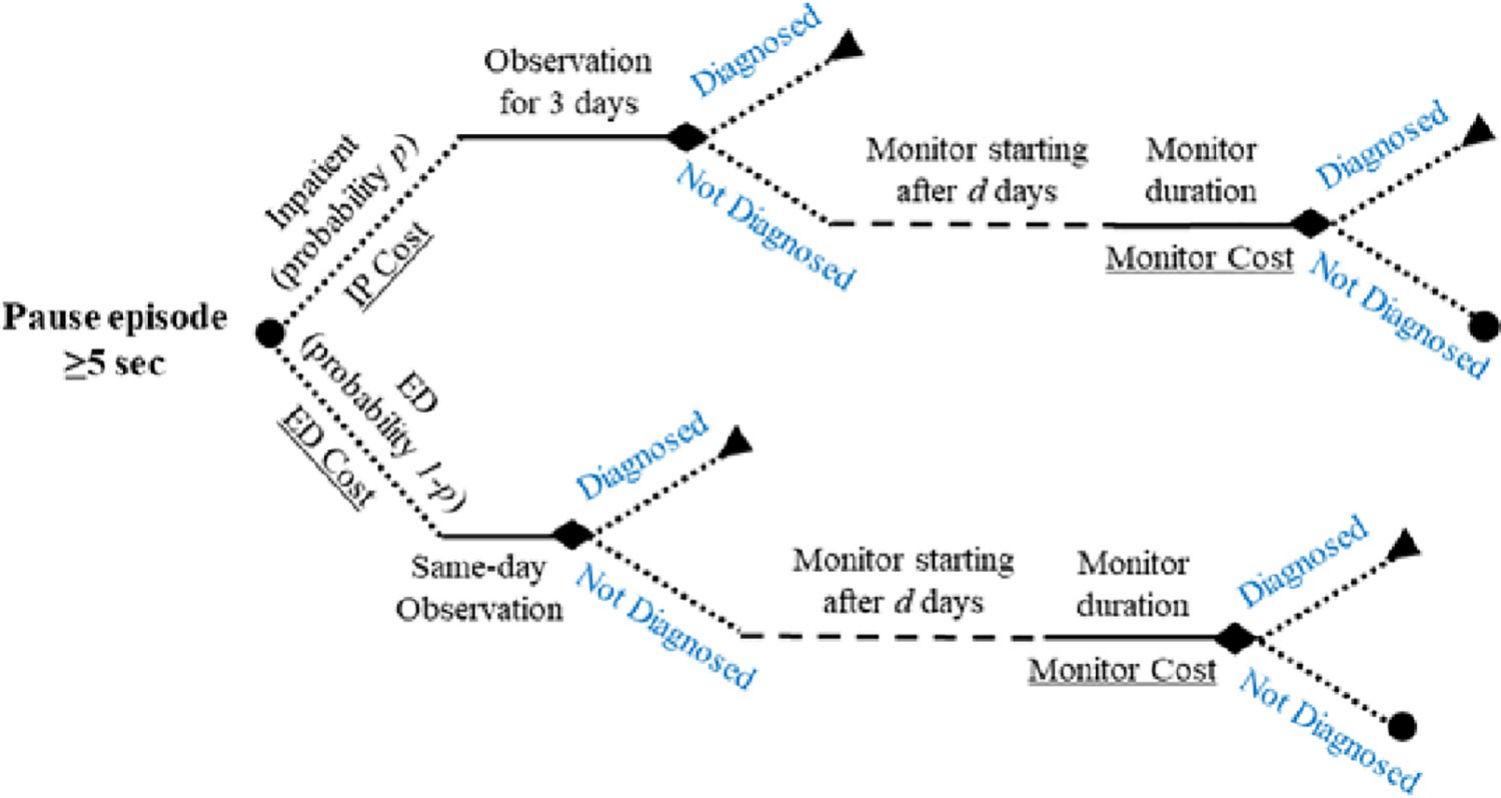
Simulation decision tree. Abbreviations: Diag = diagnosed; ED, emergency department; IP, inpatient.

The de-identified 2014–2017 Medtronic Carelink database was utilized to utilized to simulate the relative likelihood of diagnostic success with CM strategies of external ECG monitoring compared to long-term continuous ICM monitoring. Patients with ICM monitoring for unexplained syncope who were ultimately diagnosed with pause arrhythmias were randomly selected from the Carelink database, and all cardiac rhythm (ECG) data from each patient was reviewed for arrhythmic events and verified by an adjudicating committee. The occurrence and timing of arrhythmic events detected in this population was utilized to predict the rates of diagnostic success with a short-term monitor, based on the likelihood of cardiac rhythm monitoring occurring simultaneous to symptom recurrence (i.e., symptom-rhythm correlation). Diagnostic success relative to the ICM-diagnosed patients was simulated for each currently available external ECG monitor type, including: 24-hour and 48-hour Holter monitors, 14-day extended Holter, and 30-day external loop recorder (ELR), and a regimen of two sequential 30-day ELRs, with each simulation assuming that the chosen monitor modality was utilized each time symptoms recur. Fig 1 shows the decision tree that depicts the simulation. Continuous lines represent monitoring periods, model parameters are shown in italics, and cost parameters are shown underlined.

Observed pause arrhythmia events of at least 5 seconds were assumed to be symptomatic. Longer thresholds of 6 and 7 seconds were evaluated as well. The simulation begins at the first observed pause arrhythmia event. At this decision point, the simulated patient is assumed to be evaluated in the ED, with a fixed probability of 32% (*p*) [7]; otherwise the patient is admitted to the hospital for continued observation. Patients admitted to a hospital are monitored for 3 days, corresponding to the average length of stay for a syncope patient [7], while patients in the ED are monitored for the day and not admitted; if symptoms do not recur during the encounter, the patient is discharged home with an order for an external ECG monitor to be placed and begin monitoring on a randomly selected day (*d*) from a uniform distribution within day 1–7 of discharge home. If symptoms do not recur (i.e., a patient has not been diagnosed) in the simulation during the ambulatory ECG monitoring, the next symptomatic event restarts the simulation at the first decision point (inpatient admission or ED observation). A patient is considered successfully diagnosed if an assumed symptomatic event occurs while the patient is being monitored in the simulation. A patient exits the simulation once they are diagnosed in the simulation or reach end of follow-up. The relative diagnostic sensitivity from CM strategies was calculated based on the proportion of patients diagnosed by the CM strategy out of our cohort of patients who were diagnosed with ICM, after 1,000 simulations.

Health care costs related to all diagnostic evaluations were also simulated and reflect the U.S. Medicare perspective. All assumed costs related to diagnostic evaluation for syncope were included. Table 1 describes all cost values and their sources included in the simulation. Costs associated with CM strategies include the initial evaluation of the patient upon syncope recurrence in the ED or inpatient hospital, and the cost of the short-term ECG monitor device and interpretation in patients who remain undiagnosed after initial evaluation. Payer cost of initial evaluation in the ED or inpatient hospital were based on administrative claims data for patients with syncope encounters for syncope evaluation (codes ICD-10 R55 and DRG 312 Syncope & Collapse), in the absence of any codes for the treatment of any physical injury or trauma and were drawn from a log normal distribution (Table 1). Cost of short-term ECG monitors were based on average U.S. payer paid amounts. ICM-related costs include the ICM device insertion, monthly remote monitoring, in-person device check, and ICM removal, and were based on average payer paid amounts. Upon diagnosis of an arrhythmia, we assume that 80% of patients have their ICM explanted, based on our clinical experience.

**Table 1:** Simulation parameters.

Parameter	Description	Value	Distribution	Source
p	Probability of inpatient admission upon syncopal recurrence	0.32	NA	[7]
d	Days from syncope event to external monitor placement, [min, max]	[1,7]	Discrete uniform	^a^
Inpatient Cost	Syncope observation hospitalization (DRG 312), mean (SD)	$5,696 ($7,075)	Log normal	^b^
ED Cost	Syncope ED visit (ICD-10-CM R55)	$1,515 ($1,832)	Log normal	^b^
External ECG Monitor Cost	Holter monitor, 24–48 hours (CPT 93224)	$173	NA	^c^
	Extended Holter, >48 hours, up to 21 days (CPT 0295T)	$214	NA	^c^
	30-day External loop recorder (CPT 93268)	$287	NA	^c^
	30-day Mobile Cardiovascular Telemetry (CPT 93228 + 93229)	$835	NA	^c^
ICM costs	ICM Implant (APC 5222)	$6,976	NA	^c^
ICM costs	ICM remote monitoring, per month after initial implant (CPT 93298 + 93299)	$76	NA	^c^
ICM costs	ICM office visit, one year after implant (CPT 93291)	$37	NA	^c^
ICM costs	ICM Explant (CPT 33284 + APC 0020)	$746	NA	^c^

Abbreviations: APC, ambulatory payment classifications; CPT, current procedural terminology; DRG, diagnosis-related group; ECG, electrocardiogram; ED, emergency department; ICM, insertable cardiac monitor; NA, not applicable.

^a^Assumption that external ECG monitor would arrive and be placed on a randomly generated day within 7 days of discharge home

^b^ Analysis of 2017 Medicare 100% Limited Data Sets health care claims

^c^2017 U.S. National Average Medicare Payments.

The cost per diagnosis of each CM or ICM strategy was calculated as the sum of all accrued diagnostic-related costs for all patients in the cohort divided by the number of patients diagnosed in the simulation. All reported results are means and standard deviations after 1,000 simulations. Simulations were done in R using RStudio (RStudio Team, Boston, MA, USA).

## Results

**Fig 2:**
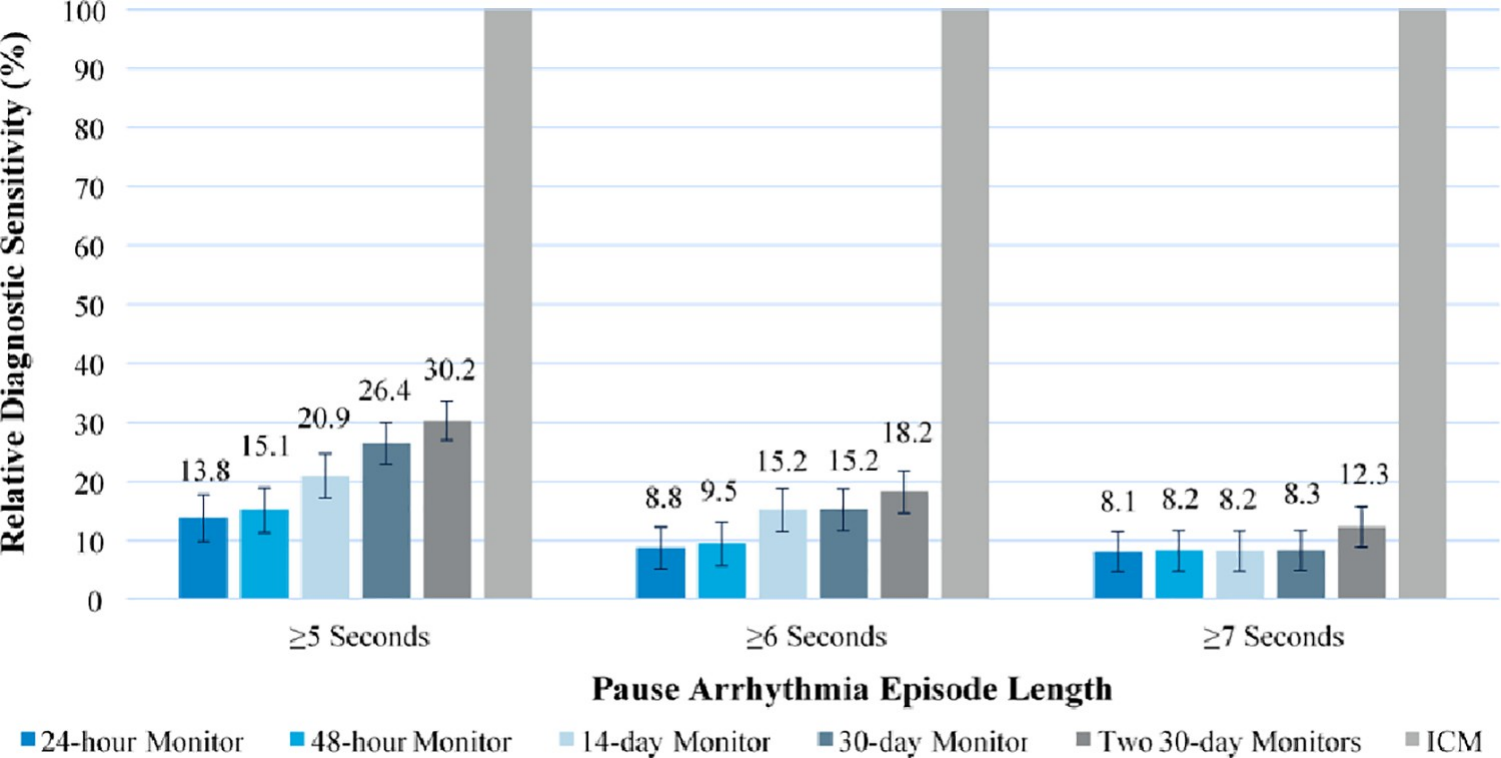
Sensitivity of conventional monitoring strategies to diagnose patients with pause arrhythmias relative to ICM. Data represent mean ± standard deviation from 1,000 simulations. Abbreviations: ICM, insertable cardiac monitor.

The cohort consisted of 44 patients with a total of 105 true pause episodes of at least 5-second duration, detected during 505±333 days of follow-up per patient (Table 2). The average age was 65.5±16.6 years, and 47.6% were male. Fewer patients experienced pause episodes with longer durations of ≥6s (n = 32), ≥7s (n = 24), or ≥8s (n = 19). Fig 2 displays the relative diagnostic sensitivity of each CM strategy compared to ICM after 1,000 simulations, stratified by the definition of pause episodes assumed to be symptomatic (definitions = ≥5, ≥6, or ≥7 seconds). For episodes ≥5 seconds, a CM strategy of using 24-hour Holters upon syncopal recurrence would diagnose on average 13.8% of patients diagnosed with ICMs. The likelihood of diagnosis increased as the duration of external monitor increased, up to a maximum of 30.2% of patients diagnosed using an approach of two consecutive 30-day monitors. The results for longer thresholds for symptomatic pause events follow a similar pattern, but due to the lower incidence of these events, the diagnostic sensitivity is lower for all CM strategies. Average days of follow-up without a diagnosis was 109 days with ICM, versus a range of 384 days (two 30-day monitor regimen) to 452 days (24-hour Holter) with CM strategies.

**Table 2:** Patient characteristics.

	Duration of Longest Pause Episode Detected during Follow-up		
	Episode ≥5s	Episode ≥6s	Episode ≥7s
N	44	32	24
Age (Mean±SD)	65.5±16.6	67.0±16.7	67.5±17.7
% Male	47.6%	56.3%	58.3%
Total Episodes in Cohort	105	53	36
Episodes per patient (Mean±SD)	2.39±2.72	1.66±1.52	1.50±1.35
Follow-up days (Mean±SD)	505 ± 333	496 ± 355	480 ± 328
Days to first event (Mean±SD)	109 ± 94	113 ± 102	103± 97

Abbreviations: SD, standard deviation.

**Fig 3:**
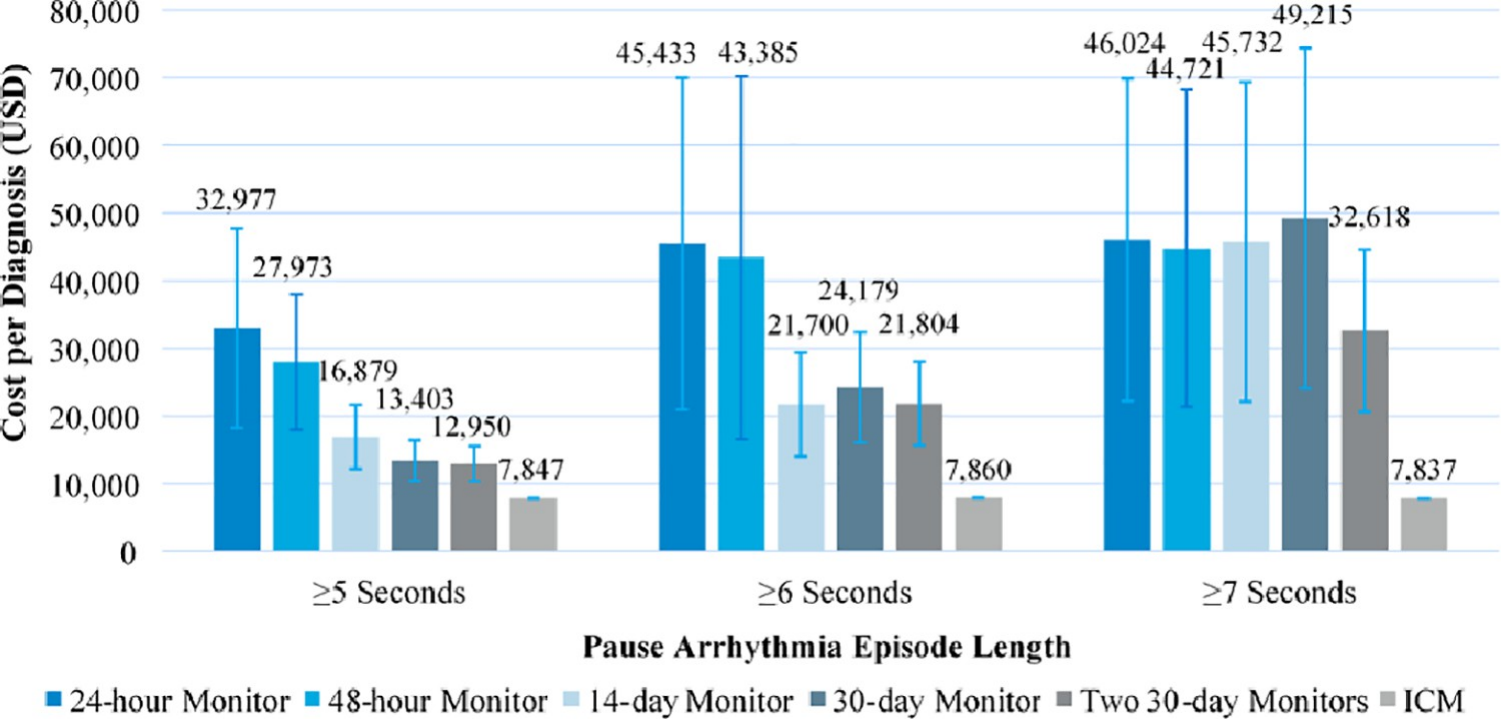
Cost per diagnosed patient with conventional monitoring strategies vs. ICM. Data represent mean ± standard deviation from 1,000 simulations. Abbreviations: ICM, insertable cardiac monito; USD, United States Dollar.

Fig 3 shows the costs per diagnosed patient from a U.S. Medicare perspective. For episodes ≥5 seconds, the mean cost per diagnosed patient for the CM strategy that uses two continuous 30-day monitors upon syncopal recurrence is $12,950±2,589. For monitoring of shorter durations, the mean cost per diagnosed patient increases, up to $32,977±14,749 for a 24-hour Holter monitor strategy. The mean cost per diagnosed patient with ICMs was $7,847. Patients with events in the longer thresholds for symptomatic pause events are costlier to diagnose with CM strategies, as episodes occurred less frequently and were thus less likely to be successfully captured via short-term monitors.

## Discussion

Our analysis found that short-term monitoring techniques have a low relative sensitivity for diagnosing pause arrhythmias compared to continuous ICM monitoring, ranging from 13.8% of diagnoses using an approach of 24-hour Holter monitoring upon syncopal recurrence, up to 30.2% utilizing two 30-day monitors. Thus, approximately 70%-86% of patients who would have been diagnosed via continuous ICM monitoring would remain undiagnosed utilizing CM strategies. The low likelihood of symptom-rhythm correlation with CM strategies may have potential long-term consequences for patient outcomes by delaying time to clinical diagnosis and treatment, as the average days of follow-up living without a diagnosis was delayed from 109 days with ICM to a range of 384–452 days with CM strategies.

Previous studies have similarly used data from implantable cardiac devices to simulate arrhythmia detection rates of various short-term external ECG monitoring techniques in non-syncope patients. A sub-analysis of the CRYSTAL AF study performed a similar analysis in patients with cryptogenic stroke and no history of AF, and found that, out of the 30% of patients in the study who were diagnosed with AF, intermittent monitoring used continuously for 7 days failed to diagnose more than 90% [16]. Similar results were found in a population with factors for both stroke and AF in the REVEAL AF study [17]. The LOOP study found that intermittent monitoring approaches become better at diagnosing AF patients when duration, dispersion, and frequency of monitoring increases, but still underperform compared to continuous monitoring with ICMs [18]. Our study extends these previous findings on AF patients to pause arrhythmia patients and confirms the evidence on the low accuracy of intermittent monitoring for cardiac diseases.

After applying payer-perspective costs to the cardiac monitoring and evaluation strategies, we found that the cost per diagnosis was higher with the CM approaches (ranging from $12,950-$32,977) compared to ICM ($7,847), driven by the low likelihood of diagnostic success with CM. While ICMs incurred a greater up-front cost, the superior diagnostic rates translated to overall diagnostic cost savings during a follow-up period averaging 505 days. As in cost-effectiveness and cost-utility analysis, cost per diagnosis is a useful metric in the coverage and clinical decision-making for monitoring and diagnostic technologies. Previous observational studies have analyzed the cost per diagnosis of different imaging and diagnostic tests in unexplained syncope patients [19]. Mendu et al. [19] performed a retrospective case review of patients presenting to the ED after a syncopal episode and analyzed the diagnostic yield and cost per test affecting diagnosis of individual diagnostic tests from a hospital cost perspective; their results show a wide range in costs per diagnosis, with the highest observed for imaging procedures (electroencephalography at $33,973 per diagnosis, and CT scans at $24,881 per diagnosis) due to the low diagnostic yield and high volume of tests ordered. Likewise, Baugh et al. [20] studied the diagnostic yields and cost per abnormal test for 11 diagnostic tests in syncope patients presenting to the ED. This study found high variability in the utilization of tests that were outside of current guidelines for the evaluation of syncope and high cost per diagnosis in the tests considered low-value in guideline recommendations, illustrating the need for closer adherence to risk stratification and evidence-based selection of a diagnostic approach to syncope as recommended [6, 21].

A structured approach to syncope evaluation utilizing dedicated syncope units and adhering to guideline-recommended evaluation practices has been shown to decrease both under-diagnosis and cost per syncope patient [22]. For example, guidelines suggest ICMs should be considered after inconclusive initial examination in patients with unexplained syncope of infrequent frequency, whereas short-term or intermittent is unlikely to be beneficial in patients with infrequent syncope [6, 21]. Our simulation builds upon the cost per diagnosis approach for a single instance of a diagnostic tool and extrapolates to an entire patient pathway to diagnosis, including repeat evaluations in syncope patients remaining undiagnosed. Results are consistent with a recent economic analysis demonstrating an overall decrease in diagnostic-related costs in patients with ICM compared to a standard of care monitoring approach [23].

## Limitations

This study has several limitations. First, the numeric patient sample size is relatively modest, however it is of high quality as it comes from a sample of patients undergoing long-term continuous ICM monitoring for unexplained syncope, randomly selected from the extensive CareLink device database and with all cardiac rhythm ECG data validated by an adjudicating committee. Due to the long duration of monitoring per patient (averaging 505 days), the cumulative monitored time of our sample is quite expansive, at approximately 22,220 patient-days. Secondly, the simulations were not able to take into account patient compliance with external ECG monitor prescribed wear-time, which has been shown in the real-world to range from 50.0% to 83.6% of prescribed days [24–28]. This likely over-estimates the likelihood of diagnostic success with CM strategies. Additionally, as linked electronic health records (EHR) were not available with the de-identified ICM device data, pause episode duration was utilized as a proxy for the occurrence of an acute syncopal episode. Since individual patients may vary in the length of pause that would be symptomatic, we display simulation results using a range of definitions generally thought to be within the range of symptomatic (i.e., episodes of ≥5-, 6-, and 7-seconds in duration). Also owing to the lack of linked EHR data, it was not possible to analyze the utilization of other diagnostic tests for syncope outside of ambulatory cardiac monitoring (e.g., tilt table test) and their relative effectiveness, or to stratify by etiology of cardiac asystolia or detailed patient history of conditions such as structural cardiac disease. Future research using device data linked to EHR would allow for interesting exploration of these topics.

As our goal was to simulate the relative effectiveness of different cardiac monitoring strategies by calculating a ‘cost per successful diagnosis,’ only patients who were indeed diagnosed with underlying arrhythmias after long-term continuous monitoring were selected, and thus our results apply to the population of patients with pause arrhythmias as opposed to the unexplained syncope population broadly. Our results are limited by the sensitivity of ICMs at detecting pause arrhythmia episodes, thus it is possible that telemetry or external ECG devices could have captured additional arrhythmias; however, as the ICM used in this study (Reveal LINQ) has a pause arrhythmia sensitivity of 99% [29] we expect this would have little effect. Finally, while by design this study does not contain separate patient cohorts which would allow a direct comparison, the simulation methodology allows us to model the potential impact of alternative interventions in a given patient population without the possible impact of confounding variables that could be found in discrete patient cohorts.

## Conclusions

Of syncope patients diagnosed with pause arrhythmias via ICM, between 70%-86% would go undiagnosed via CM strategies and therefore may not be optimally managed for syncope prevention. While the up-front cost of ICM is greater than a CM approach, the cost per diagnosis is substantially lower driven by the low likelihood of diagnostic success with CM. These findings may have implications for the selection of cardiac monitoring strategies in patients with unexplained syncope at high risk of underlying pause arrhythmias.

## Supporting information

S1 Table Relative sensitivity to diagnose pause arrhythmias and cost per diagnosed patient with conventional monitoring strategies vs. ICM. Abbreviations: ICM, insertable cardiac monitor; USD, United States dollar.
(DOCX)

S1 Data (ZIP)
