# Supplementary material for: Diagnostic sensitivity and cost per diagnosis of ambulatory cardiac monitoring strategies in unexplained syncope patients
Source: PLoS One. 2022 Jun 24;17(6):e0270398. doi: 10.1371/journal.pone.0270398 (PMC9231770; doi:10.1371/journal.pone.0270398)
Supplement: S1 Table — Abbreviations: ICM, insertable cardiac monitor; USD, United States dollar. (DOCX) [file pone.0270398.s001.docx]

**Supplemental Table 1.** Relative Sensitivity to Diagnose Pause Arrhythmias and Cost per Diagnosed Patient with Conventional Monitoring Strategies vs. ICM

| **Pause Episode Length** | **Monitor Type** | **Relative Diagnostic Sensitivity (%)**  **Mean** ± **SD** | **Cost per Diagnosed Patient (USD)** |
| --- | --- | --- | --- |
| ≥5 seconds | 24-hour Monitor | 13.8% ± 4.0 | $32,977 ± 14,749 |
|  | 48-hour Monitor | 15.1% ± 3.8 | $27,973 ± 10,046 |
|  | 14-day Monitor | 20.9% ± 3.8 | $16,879 ± 4,715 |
|  | 30-day Monitor | 26.4% ± 3.5 | $13,403 ± 3,023 |
|  | Two 30-day Monitors | 30.3% ± 3.4 | $12,950 ± 2,589 |
|  | ICM | 100% ± 0.0 | $7,847 ± 0 |
| ≥6 seconds | 24-hour Monitor | 8.8% ± 3.5 | $45,433 ± 24,515 |
|  | 48-hour Monitor | 9.5% ± 3.8 | $43,385 ± 26,815 |
|  | 14-day Monitor | 15.2% ± 3.7 | $21,700 ± 7,699 |
|  | 30-day Monitor | 15.2% ± 3.6 | $24,179 ± 8,177 |
|  | Two 30-day Monitors | 18.2% ± 3.6 | $21,804 ± 6,220 |
|  | ICM | 100% ± 0.0 | $7,860 ± 0 |
| ≥7 seconds | 24-hour Monitor | 8.1% ± 3.4 | $46,024 ± 23,821 |
|  | 48-hour Monitor | 8.2% ± 3.5 | $44,721 ± 23,429 |
|  | 14-day Monitor | 8.2% ± 3.4 | $45,732 ± 23,595 |
|  | 30-day Monitor | 8.3% ± 3.4 | $49,215 ± 25,196 |
|  | Two 30-day Monitors | 12.3% ± 3.4 | $32,618 ± 12,004 |
|  | ICM | 100% ± 0.0 | $7,837 ± 0 |

Abbreviations: ICM, insertable cardiac monitor; USD, United States dollar.
